# Infant feeding patterns before and after changes to food allergy prevention guidelines in Australia

**DOI:** 10.5694/mja2.51627

**Published:** 2022-06-30

**Authors:** Jennifer Koplin, Victoria Soriano, Merryn Netting, Rachel Peters

**Affiliations:** ^1^ Centre for Food and Allergy Research, Murdoch Children’s Research Institute Melbourne VIC; ^2^ The University of Melbourne Melbourne VIC; ^3^ South Australian Health and Medical Research Institute Adelaide SA; ^4^ The University of Adelaide Adelaide SA; ^5^ Murdoch Children’s Research Institute Melbourne VIC

**Keywords:** Infancy, Food hypersensitivity, Breastfeeding

Food allergy is an important public health problem in Australia. Randomised controlled trials have provided evidence that introducing infants to peanut and egg early reduces the risk of developing allergies to these foods.^1^ The Australian Infant Feeding Summit was therefore held in 2015 to review infant feeding recommendations.^2^ Based on a consensus among expert stakeholders (state and federal health care agencies, consumers, and experts in allergy, infant feeding, and population health), and with the aim of providing clear guideline advice to the public while balancing the need for food allergy prevention and other nutritional priorities, including the known benefits of breastfeeding, three recommendations were made: to introduce solid foods at about six months (but not before four months) of age; to introduce peanut and egg during the first twelve months of life; and to no longer use hydrolysed infant formula for preventing allergy.^2^ These recommendations were incorporated into the infant feeding guidelines of the Australasian Society of Clinical Immunology and Allergy in 2016.^3^

We have previously reported that introducing peanut during the first year of life increased in Melbourne after the new guidelines were introduced, from 28% of infants during 2007–11 to more than 88% during 2017–19.^4^ In this article, we report our assessment of breastfeeding rates, the introduction of solid foods, and the use of partially hydrolysed formula before and after the new Australian guidelines were published.

We analysed data from two population‐based studies we had undertaken using the same recruitment methods and sampling frame.^4^ Twelve‐month‐old infants were recruited at local council vaccination sessions in Melbourne, for the HealthNuts study during 2007–11, and for EarlyNuts during 2017–19. Their parents completed questionnaires including items on infant feeding practices (Box 1), with similar response rates (HealthNuts, 74%; EarlyNuts, 76%). The Royal Children’s Hospital Human Research Ethics Committee approved each study (HealthNuts, #27047; EarlyNuts, #36160).

Box 1 **Questions related to infant feeding included in the HealthNuts and EarlyNuts study questionnaires**

▪ When was solid food first introduced?
▪ Was your child ever breastfed?
▶ If yes, Is he/she still breastfed? If not still breastfed, age breastfeeding stopped.
▪ Was your child ever given formula?
▶ If yes, what formula do you use now?
▶ Type/s of formula used in the past.

The proportion of infants breastfed for at least twelve months was larger during 2017–2019 than 2007–2011 (52.7%; 95% confidence interval [CI], 50.3–55.0% *v* 35.9%; 95% CI, 34.5–37.2%), and the proportion of those who received formula slightly smaller (76.0%; 95% CI, 74.0–77.9% *v* 80.1%; 95% CI, 79.0–81.2%); changes in the proportions of infants ever breastfed or who received partially hydrolysed formula were not statistically significant. The proportion of infants introduced to solid foods at four months increased (31.9%; 95% CI, 29.7–34.1% *v* 19.2%; 95% CI, 18.2–20.4%), but not that of those introduced very early (before four months) (Box 2).

Box 2 **Infant feeding patterns in the HealthNuts (2007–11) and EarlyNuts (2017–19) studies, Melbourne**

**Table:** 

Characteristic reported	HealthNuts (2007–11)		EarlyNuts (2017–19)		
	Number	Proportion (95% CI)	Number	Proportion (95% CI)	Absolute difference (percentage points) (95% CI)
Total number of infants	5276	—	1933	—	
Age at introduction of solid foods (months)	4871	—	1740	—	
Under 4	153	3.1% (2.7–3.7%)	36	2.1% (1.5–2.9%)	–1.1 (–6.5 to 4.3)
4	937	19.2% (18.2–20.4%)	555	31.9% (29.7–34.1%)	12.7 (8.0 to 17.3)
5	1556	31.9% (30.6–33.3%)	470	27.0% (25.0–29.1%)	–4.9 (–9.6 to –0.3)
6	1974	40.5% (39.2–41.9%)	587	33.7% (31.5–36.0%)	–6.8 (–11.2 to –2.4)
7 or more	251	5.2% (4.6–5.8%)	92	5.3% (4.3–6.4%)	0.1 (–5.2 to 5.5)
Any breastfeeding	4862/5150	94.4% (93.7–95.0)	1745/1832	95.3% (94.2–96.1%)	0.8 (–0.4 to 2.0)
Duration of breastfeeding (months)	5018	—	1737	—	
Under 1	648	12.9% (12.0–13.9%)	150	8.6% (7.4–10.1%)	–4.3 (–9.5 to 0.9)
1–3	708	14.1% (13.2–15.1%)	165	9.5% (8.2–11.0%)	–4.6 (–9.8 to 0.5)
4–6	759	15.1% (14.2–16.1%)	190	10.9% (9.6–12.5%)	–4.2 (–9.3 to 0.9)
7–9	645	12.9% (12.0–13.8%)	173	10.0% (8.6–11.5%)	–2.9 (–8.1 to 2.3)
10–11	459	9.1% (8.4–10.0%)	144	8.3% (7.1–9.7%)	–0.9 (–6.1 to 4.4)
12 or more	1799	35.9% (34.5–37.2%)	915	52.7% (50.3–55.0%)	16.8 (12.9 to 20.7)
Any formula feeding	3910/4879	80.1% (79.0–81.2%)	1392/1832	76.0% (74.0–77.9%)	–4.2 (–6.4 to –1.9)
Partially hydrolysed formula used*	479/3663	13.1% (12.0–14.2%)	121/1333	9.1% (7.6–10.7%)	–4.0 (–9.9 to 1.9)

CI = confidence interval.

* For infants who received any formula during the first year of life. Some families who reported formula feeding did not report type of formula used (HealthNuts, 247 [6.3%]; EarlyNuts, 59 [4.2%]).

Breastfeeding rates were higher in the Melbourne EarlyNuts study than in the 2017–18 National Health Survey^5^ — breastfeeding ever, 95% *v* 93%; at least twelve months, 53% *v* 41% — but a smaller proportion had been introduced to solid food at six months or later (39% *v* 48%). As EarlyNuts was a food allergy study, rates of breastfeeding and the adoption of guideline recommendations among its participants may have been higher than for the general population.

Our findings suggest that introducing common food allergens early is not associated with a decline in breastfeeding. They do not establish a causal relationship between the new allergy guidelines and changes in infant feeding outcomes, but it is reassuring that the proportion of breastfed infants did not decline after their publication. Timely introduction of allergens can be recommended without concern that it might curtail breastfeeding or increase extremely early introduction to solid foods. We recommend ongoing monitoring of breastfeeding, timing of the introduction of solid and allergenic foods, and the incidence of food allergy in Australia.

## Open access

Open access publishing facilitated by The University of Melbourne, as part of the Wiley – The University of Melbourne agreement via the Council of Australian University Librarians.

## Competing interests

No relevant disclosures.
